# Antisense knockdown of pyruvate dehydrogenase kinase promotes the neutral lipid accumulation in the diatom *Phaeodactylum tricornutum*

**DOI:** 10.1186/s12934-014-0100-9

**Published:** 2014-08-09

**Authors:** Yu-Han Ma, Xiang Wang, Ying-Fang Niu, Zhi-Kai Yang, Meng-Han Zhang, Zhong-Ming Wang, Wei-Dong Yang, Jie-Sheng Liu, Hong-Ye Li

**Affiliations:** Key Laboratory of Eutrophication and Red Tide Prevention of Guangdong Higher Education Institutes, Jinan University, Guangzhou, 510632 China; Guangzhou Institute of Energy Conversion, Chinese Academy of Sciences, Guangzhou, 510650 China

**Keywords:** Microalga, Pyruvate dehydrogenase kinase, Antisense, Lipid, Biofuel

## Abstract

**Background:**

Microalgae have been an emerging biofuel resource; however, the germplasm improvement has been slow due to the lack of molecular tools. Pyruvate dehydrogenase kinase (PDK) deactivates the pyruvate dehydrogenase complex (PDC) which catalyzes the oxidative decarboxylation of pyruvate. Acetyl-CoA production via PDC is important in plant tissues that are active in fatty acid synthesis.

**Results:**

A 1261-bp cDNA of a putative *PDK* gene (*PtPDK*) was cloned from a diatom *Phaeodactylum tricornutum*, and *PtPDK* antisense knockdown transgenic diatoms were generated. Both *PtPDK* transcript abundance and enzyme activity were reduced significantly due to antisense knockdown of *PtPDK*. Neutral lipid content of transgenic diatom cells increased up to 82% as determined by Nile red staining, and fatty acid composition was not altered. Transgenic cells showed slightly lower growth rate but similar cell size with the wild type, hence retaining similar biomass productivity.

**Conclusions:**

This work first obtained a successful engineered diatom regulating a key gene involved in lipid metabolism. Our findings also provide powerful indications in enhancing microalgal lipid production by metabolic engineering for biofuel industry.

## Background

Diatoms are the main component of marine phytoplankton and play an important role in providing marine primary productivity. Particularly, a range of novel metabolic pathways have been found in diatoms which were presumably acquired during their evolution [1], so they are of major interest for the discovery of novel biological processes which are not present in other more intensively studied model organisms [2]. Microalgae have become an emerging biofuel resource [3]. Many diatom species are good candidates for biofuel production due to their high lipid content and rapid growth, such as *Phaeodactylum tricornutum*. Genetic transformation has been established in few diatoms including *Thalassiosira pseudonana*, *P. tricornutum* [4,5]. Thus, it is possible to genetically manipulate *P. tricornutum* for improving traits suited to biodiesel production [5].

The mitochondrial pyruvate dehydrogenase complex (PDC) catalyzes the irreversible oxidative decarboxylation of pyruvate to acetyl-CoA, thus directing the carbon flow into TCA cycle for yielding NADH to satisfy the energy demands of cells [6]. Thus PDC activity indicates the rate of the entry of pyruvate into the TCA cycle and subsequently to oxidative phosphorylation or fatty acid synthesis. The mitochondrial PDC, unlike its plastid counterpart, has an associated pyruvate dehydrogenase kinase (PDK) [7]. The PDC activity is primarily regulated by reversible phosphorylation by PDK, which phosphorylates and deactivates PDC [8].

In terms of the critical role of PDK in regulating PDC activity, it is important to unravel the molecular mechanisms behind the regulation. Comparative homology modeling of PDK isozymes from *Xenopus tropicalis* revealed that acetyl-CoA production via PDC is important in tissues that are active in fatty acid synthesis [9]. Overexpression of *Brassica napus* PDK in *Arabidopsis* repressed the PDC activity, leading to a decrease in seed oil content [10]. These results propose the possibility of manipulating PDK for promoting fatty acid synthesis. In the current study, we characterized a *PDK* gene from *P. tricornutum* and investigated the effect of antisense knockdown of *PDK* in *P. tricornutum*.

## Results

### Prediction and sequence analysis of putative PtPDK

**Figure 1:**
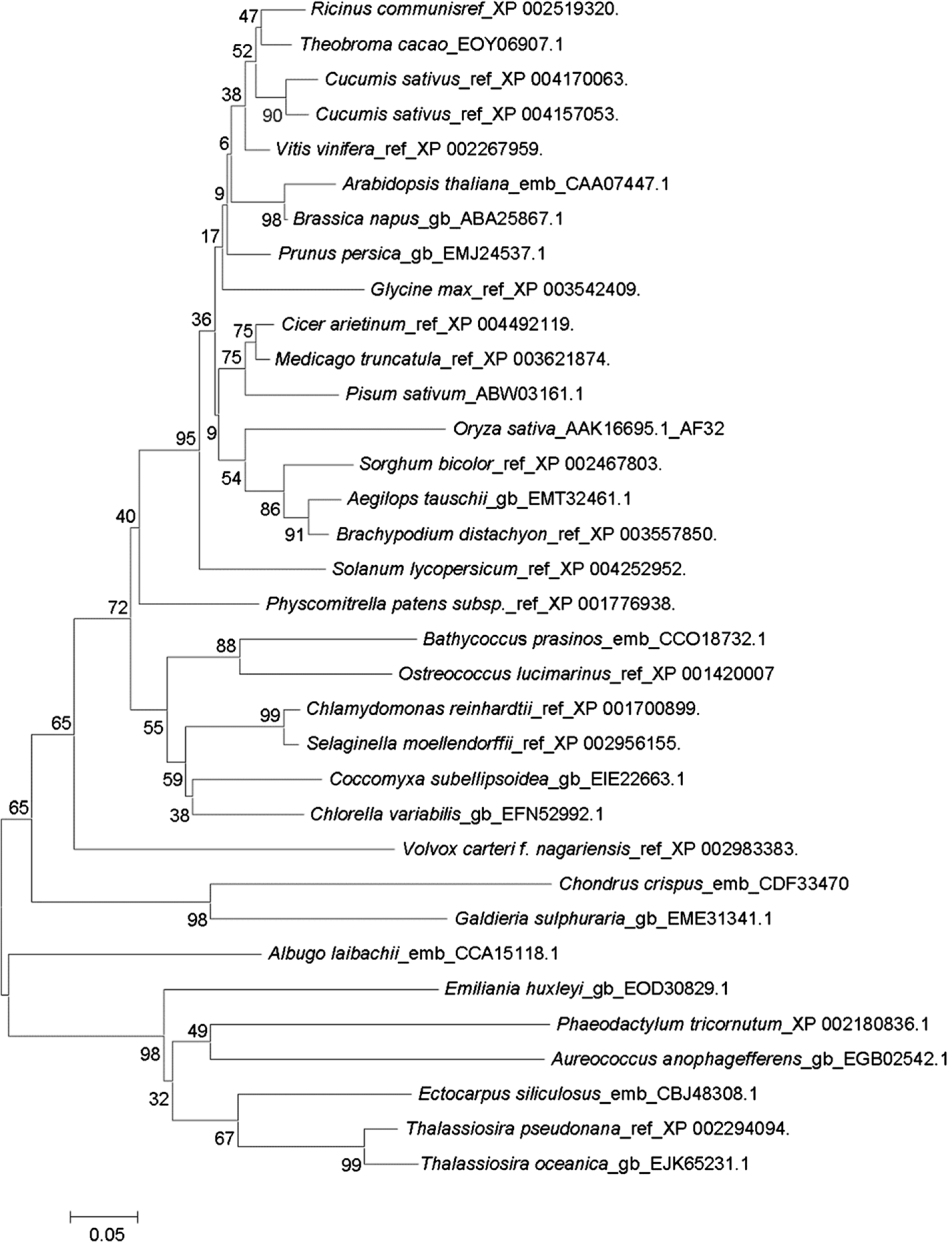
**Phylogenetic relationships of** ***PtPDK*** **with related species based on BLAST.** Amino acid sequences from various species were analyzed with software MEGA5.

Since PDK of *P. tricornutum* was not described in annotated genome sequences, homology searching was initially performed using the predicted PDK available in another sequenced diatom *Thalassiosira pseudonana* (GenBank accession: XP_002294094, THAPSDRAFT_37571) to identify the putative PDK in the genome of *P. tricornutum*. A candidate with locus tag Phatrdraft_50961 showed the highest 46% amino acid identity to PDK of *T. pseudonana* and was designated PtPDK. Amino acid sequences of other 33 species were obtained by BLAST searching in the NCBI database with the putative PtPDK. The phylogenetic tree constructed by MEGA5 demonstrated that the putative PtPDK showed high homology with PDKs from other species (Figure 1). Interestingly, PtPDK showed a close match with *Aureococcus anophagefferens*, a spherical non-motile pelagophyte with 2–3 μm diameter, which has caused ‘brown tide’ blooms in some estuaries such as northeast and mid-Atlantic US estuaries for two decades and Bohai Sea in China in recent years. They were clustered into one group with *Ectocarpus siliculosus*, a filamentous brown alga (Heterokontophyta), and two diatom species, *Thalassiosira pseudonana* and *Thalassiosira oceanica*. However, PtPDK was only weakly related to those enzymes from green algae such as *Chlorella variabilis* and *Chlamydomonas reinhardtti.* The latter actually cluster with higher plants, which is not a big surprise considering the evolution of higher plants.

### Molecular characterization of transgenic diatom by PCR, qPCR and enzyme activity assay

**Figure 2:**
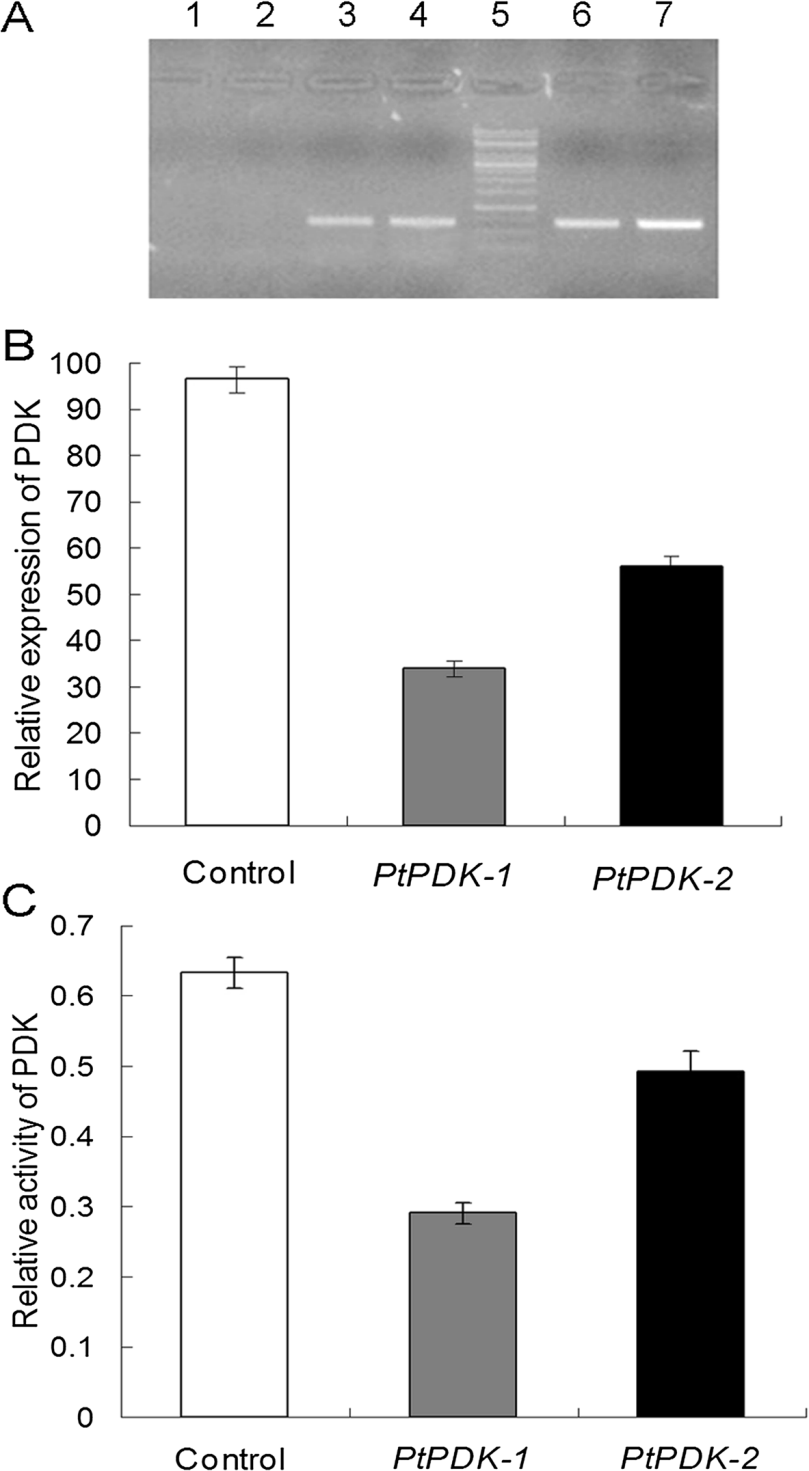
**Molecular characterization of transgenic microalgae. A)** Transgenic lines selected in liquid medium supplemented with chloramphenicol were screened by PCR using primers for CAT. A 0.7-kb CAT band was present in the transgenic lines (lane 3,4,6,7) while absent in the wild type (lane 1,2). Lane 5: 125 bp DNA ladder marker. **B)** Transcript abundance of *PtPDK* in the transgenic lines determined by qPCR. The relative expression of *PtPDK* was calculated from the Ct values of *PtPDK* mRNA using the standard curve method. Results were normalized against the *β-actin* control gene. **C)** Enzyme activity of PDK in transgenic and wild type microalgae. All assays were done in triplicates and error bars represent standard deviation.

In the final expression vector, the full-length cDNA of *PtPDK* was reversely inserted downstream of the *P. tricornutum* fcpC promoter, thus allowing the antisense expression of *PtPDK*. The surviving transformed microalgae cells were selected and subjected to PCR screening with primers for *CAT* (chloramphenicol acetyltransferase) selection marker designed in the vector. An expected 0.7-kb band was present in the transgenic lines (Figure 2A) while absent in the wild type (Figure 2A), indicating that *CAT* gene was transferred into the microalgae cells.

The transcript abundance of *PtPDK* in transgenic diatoms in the stationary phase was determined by qPCR. As shown in Figure 2B, the transgenic lines 1 and 2 exhibited a significant decrease in transcription by 65% and 42%, respectively. It implicates that the antisense knockdown had successfully repressed the mRNA expression levels of the *PtPDK* gene, while still retained some basal transcription.

The enzyme activity of PDK was further determined. As shown in Figure 2C, the relative enzyme activities of transgenic lines 1 and 2 declined by 55% and 23%, respectively, compared with that of the wild type. This result demonstrated that the suppressed *PtPDK* expression consequently resulted in a declined enzyme activity and transcript abundance showed a high correlation with enzyme activity.

### Biomass and lipid productivities of transgenic diatoms

**Figure 3:**
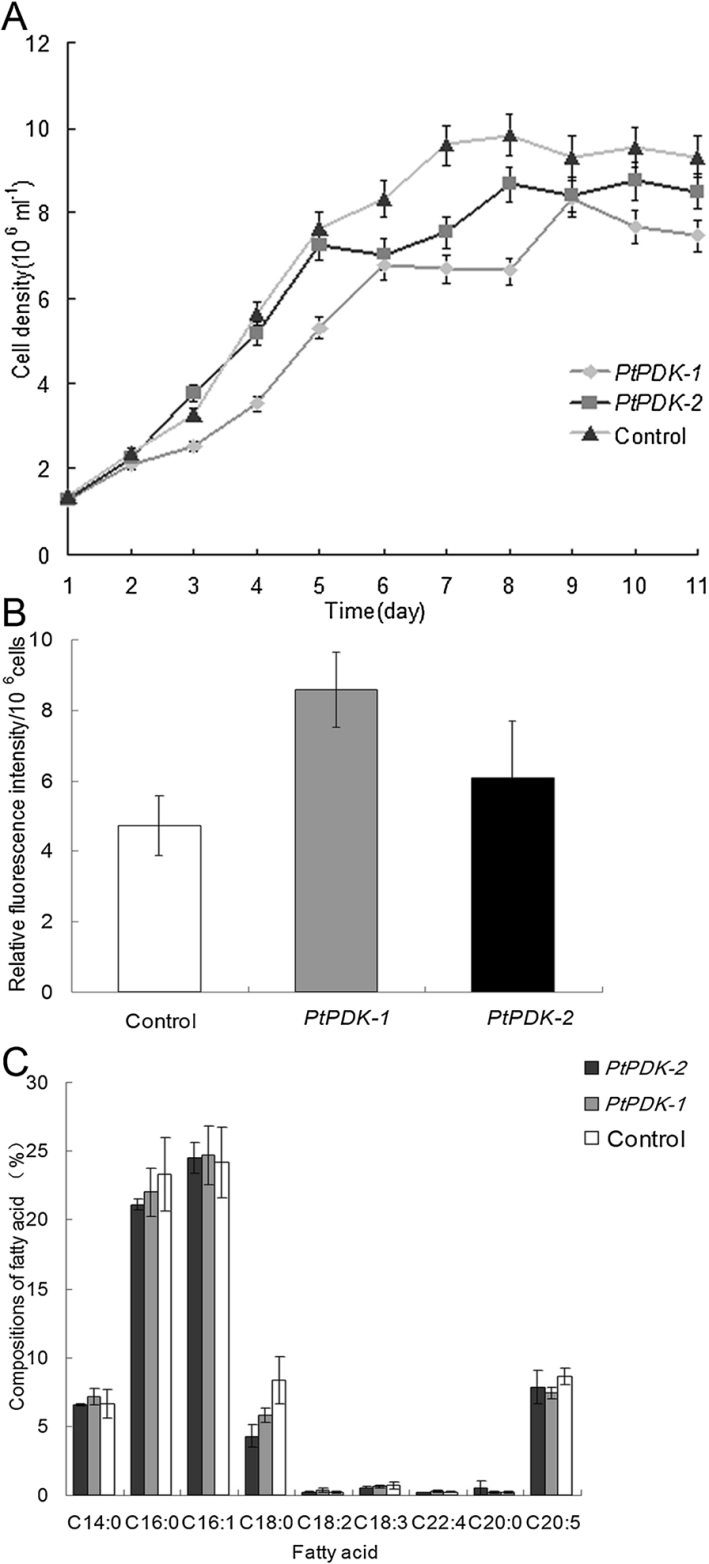
**Analysis of growth, lipid content and fatty acid profiles in transgenic microalgae. A)** Growth curves of microalgae. **B)** Lipid content per cell determined by Nile red staining. **C)** Fatty acid profiles of microalgae (weight%). All assays were done in triplicates and error bars represent standard deviation.

To determine the biomass accumulation in the antisense knockdown of *PtPDK* in *P. tricornutum*, the growth curves of transgenic lines in f/2 medium without chloramphenicol were measured (Figure 3A). The growth velocity varied in transgenic lines. Transgenic line *PtPDK-1* showed slightly lower growth rate throughout the culture cycle compared with the wild type. Transgenic line *PtPDK-2* showed a similar growth in the early phase of culture while a bit lower cell concentration in the stationary phase, compared with the wild type.

The neutral lipid content per cell indicated by the relative fluorescence intensity in Nile red-stained cultures in the stationary phase showed an increase of 82% and 33% in cells of transgenic line 1 and 2, respectively (Figure 3B). The neutral lipid content in wild type cells was initially determined to be 23.1% in dry weight, thereby the neutral lipid content in transgenic line 1 and 2 in dry weight were calculated to be approximately 42.10% and 30.76%, respectively. Hence, the antisense knockdown of *PtPDK* led to a significant increase in lipid productivity in genetically improved *P. tricornutum*.

Fatty acid profiles were further analyzed in diatom cells in the stationary phase (Figure 3C). Only a minor difference in fatty acid composition was found between transgenic and wild type algal cells which fell in the range of the standard deviation, except for C18:0 which was apparently lower in transgenic lines. These results indicate that antisense knockdown of *PDK* did not have much impact on the fatty acid composition of *P. tricornutum*.

### Morphological observation of transgenic diatom cells

**Figure 4:**
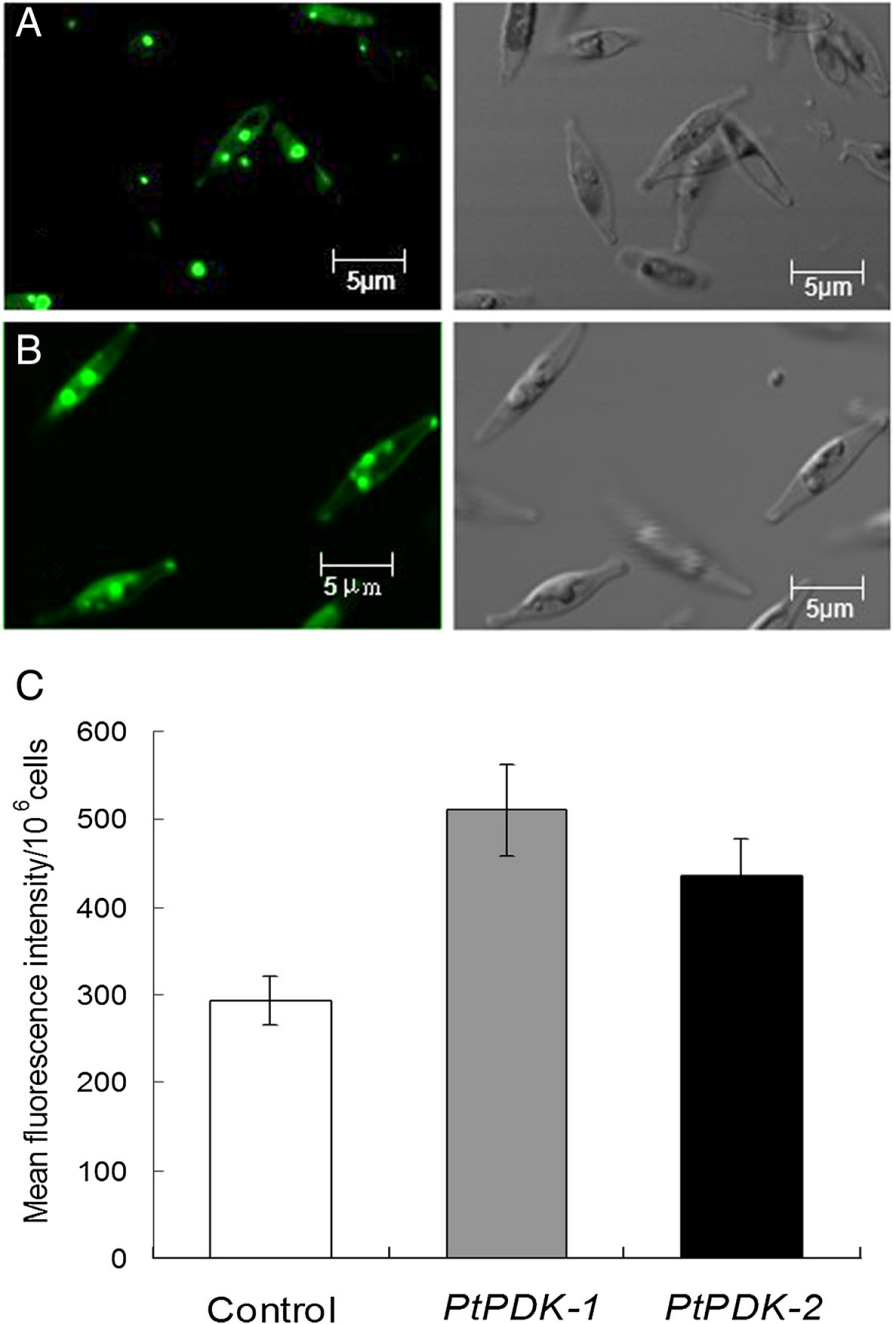
**Confocal images of** ***P. tricornutum*** **cells stained with Nile red.** Cells were photographed under confocal microscope at day 7 in the stationary phase. **(A)** wild type; **(B)** Transgenic line *PtPDK-1*. Left panel: fluorescence of neutral lipids; right panel: DIC (differential interference contrast). Bars = 5 μm. **(C)** The fluorescence of triplicate Nile red stained microalgal samples was determined by flow cytometry. The relative fluorescence intensity was calculated by subtracting the background counts.

Since the intracellular neutral lipids are mainly stored in oil bodies, the size and number of oil bodies were observed under a confocal laser scanning microscope. As shown in Figure 4, cell morphology and size remained normal compared with the wide type cells. Oil bodies in both transgenic lines showed increase in size and number, with diameters of visible oil bodies ranging from 0.2 to 2.0 μm (Figure 4B, shown are line 1). Nile red-stained diatom cells were also quantified by flow cytometry at single-cell level [11]. The relative fluorescence intensity of transgenic cells were significantly higher than the wild type, accounting for an increase of 73% in *PtPDK-1* and 49% in *PtPDK-2*, respectively, which were in accordance with the measurement of neutral lipid content in Figure 3B.

## Discussion

The current work reported a promising trial on the genetic improvement of microalgal strains for biofuel production. The primary requirement in microalgal biofuel industry is to have excellent algal species/strains. The ideal strain should have a combination of characteristics including fast growth, high oil content, strong resistance, and be suitable for large-scale cultivation. Metabolic engineering is a promising approach to achieve such an ideal microalgal strain. It is only more recently that we have the molecular tools for metabolic engineering and investigation on the complexity of changes in microalgae at molecular level. Recently, Trentacoste et al. have tried genetic engineering of the diatom *T. pseudonana* to improve lipid content by knockdown of a lipase gene [12]. The antisense strains showed an uncompromised growth comparably to the wild type, but RNAi strains showed a decreased growth. In our study, we have improved the neutral lipid contents in another diatom *P. tricornutum* by antisense knockdown of *PDK*, while the transgenic algae displayed an inconspicuously decreased growth comparably to the wild type.

Moreover, the distribution of cellular resources during the growth of microalgae is affected by the availability of light, temperature, nutrients and the amount of energy required for their use. Based on this point, for biofuel industry, the simultaneous production of biomass and oil can be addressed more rapidly by using a combination of molecular biology and environmental control in future studies.

Since de novo fatty acid synthesis occurs in the chloroplast, Roessler and Ohlrogge tried to express and target acetyl-CoA carboxylase (ACCase) to the chloroplast of the diatom *Cyclotella cryptica*. Although expression of ACCase was elevated, lipid yields did not exhibit significant changes [13]. The role of mitochondrion is usually neglected in the study of lipid biosynthesis, however, it especially plays a role in affecting the lipid synthesis through energy conversion and carbon metabolism in the cell. In seeds of *Arabidopsis thaliana*, the plastidial pyruvate kinases were important for fatty acid synthesis [14]. Although activity of PDC is directly inhibited by acetyl-CoA and NADH, which are products of aerobic oxidation of fatty acids, the major regulation on PDC is the covalent modification of PDC leading to the activated or inhibited activity [15]. PDK, a member of serine/threonine kinase family, plays a role in regulating carbohydrate metabolism by deactivation of PDC via the reversible phosphorylation of pyruvate dehydrogenase (E1) α subunit to achieve ATP-dependent inactivation of pyruvate dehydrogenase [16]. PDK has an effect on short-term regulation of pyruvate generated by glycolysis and its activity could be regulated by the molar ratios of acetyl-CoA/CoA, NADH/NAD [17].

In PDHK4 knockout mice, the promoted fat accumulation and reduced fatty acid oxidation resulted from activation of PDC by lower phosphorylation of PDC through repressing the expression of PDHK (pyruvate dehydrogenase kinase). The findings suggest PDHK4 deficiency could result in the altered upstream signaling components associated with the regulation of lipid metabolism [18]. Results of Marillia et al. provided evidence that PDK may participate in the regulation of lipid biosynthesis in *Arabidopsis thaliana* [4]. They hypothesized that enhanced mitochondrial PDC activity through antisense knockdown of *PDK* expression may lead to an increased availability of acetyl moieties converted from pyruvate which are used in the synthesis of storage lipids in the developing seed. This hypothesis is consistent with the metabolic fluxes determined in developing embryos of *Brassica napus*, in which mitochondrial flux largely contributed to the provision of precursors for cytosolic fatty acid elongation [19].

However, the study on PDC in fatty acid biosynthesis in microalgae has not been reported. Considering that complete blocking of gene expression might be harmful to the cell and antisense knockdown usually achieves partial blocking, thus, in this work, antisense knockdown of *PDK* was adopted to develop genetically engineered microalgae suitable for industrial application. In this study, we demonstrated that effective knockdown of *PtPDK* expression resulted in increased lipid content without compromising biomass much. Transgenic cells showed similar growth as well as cell size with the wild type, therefore ensuring the biomass productivity. Results also implicated that PDK plays an important role in storage lipid accumulation through the regulation of mitochondrial carbon flux. Though the mere step of antisense knockdown of *PtPDK* has not promoted overall lipid yield enough to achieve an “ideal” industrial strain, the trial here has paved the way for microalgal biofuel development through the approach of metabolic engineering.

## Materials and methods

### Microalga and culture conditions

Microalga strain *Phaeodactylum tricornutum* was obtained from the Freshwater Algae Culture Collection of the Institute of Hydrobiology, China (No. FACHB-863). Microalgae were grown as batch cultures in flasks containing f/2-Si medium (omitting Na_2_SiO_3_ · 9H_2_O). Seawater taken from the Gulf of Dayawan was filter-sterilized through 0.22 μm filters and amended with f/2 nutrients and used as medium. Cultures in liquid medium or on the plate were grown at 21 ± 1°C in an artificial climate incubator. Cool-white fluorescent tubes provided an irradiance of 200 μmol photons m^−2^ s^−1^ under a 12/12 h light/dark regime. Growth curve was determined by counting cells with Brightline hemocytometer under a microscope every day.

### Cloning and analysis of putative *PtPDK* gene

The 1,261-bp full-length coding region of putative *PtPDK* was amplified from total RNA of *P. tricornutum* by reverse transcription PCR with primers: 5′-ACCATGGAATTCGGAAATGTGAAACC-3′ and 5′-GTAATTGGCACGGGTTCACTTGTA-3′ flanking the two ends of *PtPDK* cDNA. The amplified cDNA sequence was confirmed by sequencing analysis at both orientations. Amino acid sequence alignment and similarity among species were determined using ClustalX version 1.83 and BLAST on NCBI (http://blast.ncbi.nlm.nih.gov/Blast/). Phylogenetic tree of protein clusters from various species was constructed by neighbor-joining (NJ) method using software MEGA 5 [20]. Subcellular localization of *PtPDK* was predicted by softwares SignalP 4.1 (http://www.cbs.dtu.dk/services/SignalP/) and iPSORT (http://ipsort.hgc.jp/predict.cgi) online.

*PtPDK* was cloned into a *P. tricornutum* transformation vector modified from a previously constructed plasmid pHY11 [21]. In the final vector designated pHY18-antiPDK, *PtPDK* was cloned between *fcpC* promoter and *fcpA* terminator of fucoxanthin chlorophyll a/c binding protein gene of *P. tricornutum*. Chloramphenicol at the final concentration of 250 μg/ml was used as selection marker in transformed *Escherichia coli* and *P. tricornutum* cells. *P. tricornutum* was transformed with plasmid pHY18-antiPDK by electroporation using Bio-Rad GenePulser Xcell apparatus (Bio-Rad, USA) following the protocol of Niu et al. [21].

### Molecular analysis of transgenic microalgae by genomic PCR and qPCR

The incorporation of constructed gene expression cassettes into diatom genomes was demonstrated by genomic DNA PCR. Genomic DNA was extracted using Universal Genomic DNA Extraction Kit Ver.3.0 (Takara). PCR was performed with primers of *CAT* gene including forward primer (5′-ATGGAGAAAAAAATCACTG-3′) and reverse primer (5′-TAAGCATTCTGCCGACAT-3′).

Transcriptional abundance of *PtPDK* was determined by quantitative real-time PCR (qPCR) performed in Boxin Co., Guangzhou, China. Total microalgae RNA was extracted from the microalage cells using an E.Z.N.A.™ Plant RNA Kit (Omega Bio-Tek) and reverse-transcribed using an Omniscript reverse transcription kit (QIAGEN) with random hexamer primers [22]. Reactions were performed in 96-well optical reaction plates with 20 μl mixture per well, using a SYBR Green Kit (Takara) and a 7300 Sequence Detection System (Applied Biosystems) following the manufacturers’ instructions. Primers used for *PtPDK* were (5′-ACCATGGAATTCGGAAATGTGAAACC-3′) and (5′-GTAATTGGCACGGGTTCACTTGTA-3′) which are unique to *PtPDK* sequence. β-actin was used as a housekeeping marker control and primers were ACT1f (5′-AGGCAAAGCGTGGTGTTCTTA-3′) and ACT1r (5′-TCTGGGGAGCCTCAGTCAATA-3′). The Ct (threshold cycle) for each well was measured. *PtPDK* mRNA accumulation in transgenic and wild type cells were quantified after normalization to β-actin.

### Neutral lipid content analysis

The dye Nile red has been used to detect the intracellular lipid droplets by fluorescence microscopy and quantified with a photometer and photomultiplier connected to the microscope [23]. Nile Red (Sigma) staining was performed following Yang et al. [24] to detect the cellular neutral lipid contents of *P. tricornutum*. Thirty microliters of Nile red (0.1 mg/ml acetone solution) was added into a 3 ml aliquot of cell culture in triplicates, then mixed by rapid inversion and incubated in darkness for 20 min at room temperature. The stained cell cultures were transferred to a 96-well plate and cellular fluorescence intensity was quantitatively determined with a microplate reader Hitach F4600 (Hitach, Japan). The wavelengths used were 530 nm for excitation and 592 nm for emission. The relative fluorescence intensity values could provide quantitative comparison of neutral lipid contents between the cell cultures. For dry weight determination, neutral lipid content in *P. tricornutum* cells was initially measured by gravimetric method. 20 mg lyophilized microalgae were mixed with 2 ml methanol, 2 ml chloroform, and 1 ml 5% NaCl by vortex for 2 min. The mixture was centrifuged at 8000 g for 4 min and the chloroform layer was collected. The same extraction procedure was repeated three times and the combined extracts were dried by N_2_ flow. Then the lipid residue was dried in oven at 60°C and weighed to yield dry weight.

### Fatty acid composition analysis

Total lipids were extracted according to the protocol from Yang et al. [24] which was modified from Lepage et al. [25]. In particular, transgenic and wild type cells in the stationary phase (250 ml each) were harvested for lipid extraction. Fatty acid composition was determined based on fatty acid methyl ester (FAME) by gas chromatography–mass spectrometry (GC-MS) at Guangdong Institute of Microbiology, China. A 30 m × 0.25 mm × 0.25 μm DB-5 quartz capillary column was used. Fatty acids were identified with the equipped NBS spectrum library. Integrated peak areas were calculated by normalization to acquire the relative contents (percentage of weight).

### Morphological observation of transgenic diatom cells

To visualize the morphological changes of transgenic cells and particularly oil bodies, Nile red (0.1 mg/ml in acetone) was added into cultured cells at a 1:100 ratio and placed in darkness for 10 min. Cells were observed under a laser-scanning confocal microscope LSM510META (Zeiss), with excitation wavelength of 525 nm and emission wavelength of 550–570 nm. Images were captured randomly from at least 20 visual fields per sample, and typical cells are presented here.

### Measurement of enzyme activity of PDK

Total proteins were extracted from transgenic and wild type algal cells for enzyme activity analysis. Enzyme activity of PDK was measured by using a Pyruvate dehydrogenase kinase kit (Jiemei, China). Relative activities of PDK were represented by the production of NADH (μmol/min per mg protein from algal cells) measured by absorbance [26].

### Nile red stained diatom cells assayed by flow cytometry

Flow cytometry is used as a rapid approach for qualitative and quantitative analysis of individual cells which can detect a variety of biochemical reactions accurately in cells [27], especially analysis of large number of individual cells by fluorescence staining [28]. It has been used to measure the fluorescent properties of individual algae cells and the fluorescence intensity reflects the lipid content of stained cells [29]. In this work, 3 ml aliquot of cell culture in the stationary phase in triplicate were stained with Nile red (0.1 mg/ml acetone solution) as above, and then analyzed using a FACS-Aria microflow cytometer (Becton Dickinson, USA). The wavelengths used were 530 nm for excitation and 592 nm for emission.
